# Assessing the Acceptability, Feasibility and Sustainability of an Intervention to Increase Detection of Domestic Violence and Abuse in Patients Suffering From Severe Mental Illness: A Qualitative Study

**DOI:** 10.3389/fpsyt.2020.581031

**Published:** 2020-10-28

**Authors:** Roos E. Ruijne, Astrid M. Kamperman, Kylee Trevillion, Carlo Garofalo, Mark van der Gaag, Milan Zarchev, Stefan Bogaerts, Louise M. Howard, Cornelis L. Mulder

**Affiliations:** ^1^Department of Psychiatry, Epidemiological and Social Psychiatric Research Institute, Erasmus University Medical Centre Rotterdam, Rotterdam, Netherlands; ^2^Section of Women's Mental Health, King's College London, Institute of Psychiatry, Psychology and Neuroscience, London, United Kingdom; ^3^Department of Developmental Psychology, School of Social and Behavioral Sciences, Tilburg University, Tilburg, Netherlands; ^4^Fivoor Science and Treatment Innovation, Poortugaal, Netherlands; ^5^Parnassia Academy of the Parnassia Psychiatric Institute, The Hague, Netherlands; ^6^Department of Clinical Psychology and Amsterdam Public Mental Health Research Institute, VU University, Amsterdam, Netherlands; ^7^Antes, Department of the Parnassia Psychiatric Institute, Rotterdam, Netherlands

**Keywords:** domestic violence and abuse (DVA), psychiatric patients, education, public mental health care, qualitative study, community mental health agencies, outpatient care

## Abstract

**Rationale:** Despite interventions to improve detection rates, domestic violence, and abuse (DVA) remains largely undetected by healthcare services. We therefore aimed to examine the acceptability, feasibility, and sustainability of an intervention aiming to improve DVA detection rates, which included a clear referral pathway (i.e., the BRAVE intervention) and to explore the acceptability and feasibility of DVA management and referrals in general, in the context of low detection rates.

**Methods:** Qualitative study design with four focus groups of 16 community mental health (CMH) clinicians from both control and intervention arms. The focus groups discussed managing DVA in clinical practice and staff experiences with the BRAVE intervention in particular. Focus groups continued until saturation of the subject was reached. Interviews were analyzed using a thematic analysis approach.

**Results:** DVA was seen to be highly relevant to mental healthcare but is also a very sensitive subject. Barriers in CMH professionals, institutions, and society meant CMH professionals often refrained from asking about DVA in patients. Barriers included communication difficulties between CMH professionals and DVA professionals, a fear of disrupting the therapeutic alliance with the patient, and a lack of appropriate services to help victims of DVA.

**Conclusion:** The BRAVE intervention was acceptable but not feasible or sustainable. Personal, institutional, and public barriers make it not feasible for CMH professionals to detect DVA in mental healthcare. To increase the detection of DVA, professional standards should be combined with training, feedback sessions with peers and DVA counselors, and routine enquiry about DVA.

**Clinical Trial Registration:** ISRCTN, trial registration number: ISRCTN14115257.

## Introduction

Domestic violence and abuse (DVA) are important societal problems with negative consequences for individuals and for society. DVA is defined as “any incident of threatening behavior, violence or abuse (psychological, physical, sexual, financial, or emotional) between adults who are or who have been an intimate partner, friend, family member or otherwise closely related person (e.g., caregiver or roommate)” (1). Although prevalence is high across all layers of society, certain groups—such as severely mentally ill (SMI) patients—are more vulnerable to being victims of DVA (2, 3). In this article we define severe mental illness as any mental disorder resulting in serious functional impairment, which causes significant limitations in daily activities (4). DVA victimization can have serious short and long-term health mental and physical consequences (5–7) and has a prevalence of around 20% in SMI patients (8). Compared to the general population, SMI patients are also more often a perpetrator of DVA (2% in the general population vs. 7 to 8% in persons with SMI) (9, 10). However, SMI patients are still more often a victim of DVA than a perpetrator of DVA (11). In this article we therefore focus on victims of DVA. Despite this high prevalence of DVA and the risk of serious consequences, mental healthcare professionals detect only a fraction of cases (12). Many of the barriers to inquiring about DVA and to detect DVA in psychiatric patients are related to professional shortcomings such as a lack of knowledge and confidence in the interview skills needed (13). A cluster randomized controlled trial (RCT) called the IRIS (Identification and Referral to Improve Safety) study (14) including a training sessions, a prompt in the medical record to ask about abuse, and the establishment of a referral pathway-reported that primary care providers' shortfalls in DVA knowledge and skills can be successfully addressed using a training program. This intervention increased DVA detection rates and number of referrals to DVA services. Promising effects were also demonstrated by the subsequent adaptation of this training for mental healthcare providers (Linking Abuse and Recovery through Advocacy, LARA (15), which formed the basis in the Netherlands for a cluster randomized controlled trial (RCT) called BRAVE (Better Reduction trough Assessment of Violence and Evaluation) (16). The BRAVE training comprised of three elements: training for mental health care professionals on DVA; training on mental health for DVA professionals, and the provision and establishment of a referral pathway between community mental health (CMH) services and DVA services for SMI patients who were victims of DVA. Although the results of the RCT showed that the intervention was followed by a significant improvement in DVA knowledge and management skills, and by a change in attitudes toward DVA among mental healthcare professionals, the number of DVA cases detected did not increase (Ruijne et al., in review, Journal of Interpersonal Violence). The purpose of this parallel qualitative study was to (1) explore the acceptability, feasibility and long-term sustainability of the BRAVE intervention and (2) to explore the acceptability and feasibility of DVA management and referrals in general, with a focus on: knowledge about DVA, assessment of DVA, safety, and treatment/follow-up. The intervention we used in our cluster randomized controlled trial was new and has not been used in research before. During the intervention period we used quantitative methods to assess the intervention (data not reported here). However, we also wanted to gain more in-depth knowledge on the motives and behavior of CMH professionals on their decision to discuss DVA or not discuss DVA. For this purpose, we deemed a qualitative approach to be the best suited method. We hypothesize that perhaps it is not feasible or acceptable to discuss DVA with patients for reasons unknown for us. With the assessment of the “feasibility” and “acceptability” in both control CMH professionals as professionals working in intervention teams, we wanted to assess whether it is feasible and/or acceptable to discuss DVA patients and what the underlying reasons could be tot not assess DVA, irrespective of the intervention. To gain more variety in the gained information from interviews, we decided to interview members from control teams as well as intervention teams.

## Methods

### Design

Four focus groups were conducted with CMH professionals working in teams participating in the BRAVE study. During the 12 month follow-up period of the trial, we also used researchers' field notes to collect context information. Reporting of this article follows the COREQ (Consolidated criteria for reporting qualitative research) guidelines (17).

### Study Setting

The BRAVE study consists of a qualitative component and a quantitative component. The quantitative component is a cluster RCT, which aimed to improve the detection and referral rates of SMI patients who are a victim of DVA using the BRAVE-intervention (ISRCTN 14115257). The intervention used in the BRAVE study consisted of three parts; (1) training in DVA knowledge and skills for mental health professionals in community mental health (CMH) teams, (2) training in mental illness and mental healthcare services for DVA professionals, and (3) the provision and establishment of a direct-care referral pathway between CMH services and DVA services for victims of DVA with SMI. To increase referral rates of patients who are victims of DVA we also aimed to: (1) provide quick access to DVA services, and (2) manage the expectations of CMH professionals and DVA professionals by providing information about the possibilities and limitations in helping DVA victims for both mental healthcare providers and DVA services. Unfortunately, due to reorganizations within the DVA services during the study period, we were unable to provide a training to manage expectations on mental health care for all DVA professionals. The trial was conducted in an urban area of the Netherlands in two municipalities: The Hague and Rotterdam. Mental healthcare in both municipalities is provided by two CMH institutions, namely BAVO Europoort (Rotterdam) and Parnassia (The Hague). Both institutions provide outpatient and inpatient mental healthcare and cover the Rotterdam-Rijnmond and The Hague regions which in total have ~ 2.5 million inhabitants.

The trial included 24 CMH teams that generally consist of around 10 professionals: psychiatric nurses, psychologists, psychiatrists and social workers. The majority of these teams consist of general nurses and/or psychiatric nurses. Twelve teams were randomized to the intervention condition and twelve to the control condition. The CMH teams in the intervention condition received a training of about 8 h, details regarding the intervention can be found in the protocol paper (16) and the quantitative paper of the BRAVE RCT (*Ruijne et al., in review, Journal of Interpersonal Violence*). The CMH teams in the control condition provided care as usual. The BRAVE intervention used a naturalistic approach and consisted of more elements than a training. The BRAVE intervention offered tools (i.e., conversation techniques, safety checklist, and memory aids) in helping to assess and manage DVA and participants were encouraged to use these tools throughout the intervention period. However, apart from the Meldcode protocol, the intervention did not consist of a mandatory, predefined method to assess and refer victims of DVA. Ethical approval for the BRAVE trial (both the trial and the qualitative study) was obtained from the Medical Ethical Committee at Erasmus University Medical Center, (MEC-2015-409) on June 10th, 2015. The quantitative results from the BRAVE RCT are described in detail elsewhere (Ruijne et al., submitted).

### Context of DVA Services

Municipalities in the Netherlands have their own DVA services, each offering various types of care, ranging from shelters to empowerment courses. However, as part of a nationwide program of municipal reorganizations in 2015, an umbrella organization called Veilig Thuis (i.e., “Safe at Home”) was founded. Veilig Thuis is intended to be an organization that professionals can consult for advice, or where they can report DVA or child abuse. Reporting a case is mandatory only when there is immediate or recurring danger. Veilig Thuis must investigate the individual case and refer the victim to the appropriate DVA service. Although Veilig Thuis does not take victims into their care, it does function as a gatekeeper and case manager for individual cases. Veilig Thuis is embedded in the so-called Meldcode protocol, a guideline for referring victims of DVA (18), the Meldcode is solely used by other institutes than Veilig Thuis. The Meldcode consists of five steps: (1) assessment of DVA signs, (2) consultation with a direct colleague or Veilig Thuis, (3) discuss DVA with the victim, (4) consider all available information gathered in the first three steps, decide if the patient is a victim of DVA and assess the safety of the situation, and (5) in this final step, the healthcare professional has to make two decisions, namely: should the case be reported to Veilig Thuis; and/or could suitable care be provided within their own organization. Following the steps of the Meldcode protocol is mandatory, but reporting DVA cases to Veilig Thuis is not. The BRAVE study was conducted at the same time as the nationwide reorganization of the municipalities and the introduction of Veilig Thuis. Due to this reorganization, however, Veilig Thuis was not fully functional at the start of the BRAVE study, and could not offer all previously announced services.

### Participants

All CMH professionals involved in the BRAVE study were eligible for inclusion in the four focus groups. They were actively recruited through e-mail, newsletters, and during team visits. After the intervention period, we sent bi-weekly invitations to all representatives of the participating CMH teams until we achieved a minimum of three and a maximum of five participants per focus group. To ensure maximum information density and saturation, two focus groups consisted of CMH professionals employed by mental health institutions situated in the Rotterdam Rijnmond region and two focus groups consisted of CMH professionals employed by the mental health institution situated in region of The Hague. Together, these mental health institutions are the largest conurbation in the western Netherlands. One of these groups consisted entirely of CMH professionals who received the BRAVE intervention (*Ruijne et al., in review, Journal of Interpersonal Violence*), and the other of CMH professionals in the control condition. The other two focus groups comprised a mix of participants from the intervention and control conditions. We also used mixed focus groups to be able to benefit from the discussion between participants of the participants of the intervention teams and the control teams. With using mixed focus groups, we expected a wider scope of views on the subject of DVA and different types of information as compared to when we would have had homogeneous focus groups. To compose the focus groups, we used convenience sampling. All participants provided written informed consent in advance and could withdraw from the study at any time. The protocol and addendum were approved by the Medical Ethical Committee of the Erasmus Medical Center (MEC 2015-409). All participants received an information letter regarding the process of the study beforehand and signed a consent form. This study was conducted in accordance with the Declaration of Helsinki (1964), as amended in Edinburgh (2000).

### Procedures

#### Focus-Group Discussions

The BRAVE intervention started February 2016 and ended in February 2018. Each of the four focus groups were held at different locations after the end of the intervention: in October 2017 for the Rotterdam Rijnmond site and in October 2018 for the The Hague site. Each was held in a neutral meeting room at the participating institutions. Two weeks in advance, participants received an information letter including the purpose of the interview. To ensure attendance, a reminder was sent 1 week later. Each focus group lasted ~ 3 h, including a half-hour break. The discussions were led by two researchers: RR, who acted as a moderator, and a research assistant, who monitored the process and intervened if necessary, such as when probing for details or to ensuring that all predefined themes had been discussed. The moderator was not part of the BRAVE intervention itself, but did help develop the BRAVE training. Two independent trainers provided the training to the teams. The moderator was assisted by an independent research assistant. The transcripts were analyzed by the research assistant and the moderator. The interrater reliability was secured with the help of AK. Additionally, the focus groups were both conducted before the results of the RCT of the BRAVE intervention were known, minimizing the potential bias of the moderator. Each group started by introducing all participants, followed by an informal question to introduce the main theme (DVA). The subsequent questions followed a semi-structured approach and were related to themes defined a priori: the acceptability, feasibility and sustainability of the BRAVE intervention (intervention teams) or DVA management in general (control teams), knowledge about DVA, assessment of DVA, safety, and treatment/follow-up of DVA). After each focus group session, all initial thoughts and ideas the researchers conducting the focus group session had were documented. From these notes themes arised, they were added to the topic list and included in the subsequent focus groups. The discussions were audio recorded and transcribed verbatim.

#### Context Information

We recorded information on participants' overall experiences with referral sites, the intervention and cases of DVA during the study period. This contextual information, which gave us more detailed insight into daily work routines, was collected during training days, after interviews, and during site visits of all included teams. It was used to formulate questions and core themes to use during the focus groups.

### Data Analysis

The data was analyzed using a thematic analysis approach, a flexible, analytic method that is widely used in qualitative health services research (19, 20). The analysis consisted of four steps; First, the transcripts were coded for the predetermined themes: acceptability, feasibility and sustainability of the BRAVE intervention and the feasibility of the management and referral of DVA for both intervention and control teams, while also making notes and use open coding for possible themes not directly related to the subject. A researcher trained in qualitative research (RR) conducted the initial coding. Second, after initial coding of the predetermined themes, we used open coding to find more specific information and to find emerging themes, subthemes, or patterns that were not driven by the initial research questions, but still relevant to the interpretation of the study results. Third, we applied structure and hierarchy to distinguish between general themes and detailed sub-themes. Fourth, all codes were reviewed and aggregated according to the themes deemed appropriate and subsequently labeled accordingly. Interrater–reliability was assessed on a theme level per 5-line block of the transcript. In total, 15% of the full transcripts was coded by RR and AK independently. For the purpose of calculating Interrater reliability we used the information on whether the text coded for a theme and which theme (acceptability, feasibility or sustainability). The Kappa level was 0.85 with a 95% confidence interval of 0.81–0.88. Nvivo 11 software (21) was used to code and analyze the data. When reporting the results, quotes from respondents are used to illustrate the results.

## Results

### Characteristics of the Participants

In total, 16 mental healthcare professionals participated in the focus groups, which comprised between three and five participants, of whom 63.5 % (10/16) were female and 37.5% (6/16) of whom had received the BRAVE intervention. The groups consisted of five social psychiatric nurses, three general nurses, four social workers, three psychologists, and one psychiatrist. Participants' age varied from 25 to 56 years (M = 41;SD = 10.0). Years of experience working in mental healthcare ranged from 1 to 27 (M = 11; SD = 7.3). All results are derived from the transcripts of the focus group discussions.

### Key Themes

Supplementary Table 1 shows the key themes that were identified prior to the start of the focus groups and the themes derived from the focus groups discussions. The table is divided into two halves: the upper half contains all key themes and sub themes from participants from intervention teams related to the BRAVE intervention and the lower half contains all themes from participants from both intervention teams as well as control teams.

### Mental Healthcare Professionals Working in Intervention Teams

#### Acceptability of the BRAVE Intervention

Participants in the BRAVE intervention were asked to reflect on the intervention's training sessions, which they evaluated positively, referring to most mentioned beneficial elements were: (1) the training had provided sufficient time for practice and interaction; (2) because the training content was pragmatic in nature, knowledge and skills could be implemented immediately in clinical practice; (3) participants preferred physical attendance to e-learning modules; (4) the trainer had good understanding of the participants' work- and expertise with regard to DVA.

However, participants in the BRAVE training would have liked more information on the practical aspects of healthcare professional -patient confidentiality, patient autonomy and duty of care, and how to balance them.

“*I now think I'd like to have heard more about healthcare professional-patient confidentiality and your duty to provide proper care. The training covered it a bit, but there should have been more about it.”* [Intervention team, male, nurse (on the training)]

Participants said that they wanted more practice with what they could and could not do within the legal framework of healthcare professional–patient confidentiality. Even though the training did cover this, participants felt that in practice, they often do not feel confident enough in their knowledge. One participant said he wanted another course that specifically dealt with this topic.

When asked to describe the characteristics of an ideal trainer, participants indicated that he or she should be able to impress, motivate and enthuse you. A participant from a control team who attended a mixed focus group said that personal stories, such as DVA experiences told by DVA survivors had helped to become sensitive to DVA experiences. Participants from intervention teams, who also heard personal stories during the training, agreed with her statement.

“*Hearing about the victims' experiences helped me a lot. During the training on DVA, one of the victims told the story of the abuse she'd endured from her mother. That had a real impact on me—it made me more alert, and it made me think.”* [Control team, female, social worker]

#### Feasibility of the BRAVE Intervention

All participants who had received the BRAVE intervention found it to be feasible, they also used the learned skills and knowledge on DVA in their daily routine. The BRAVE intervention provided tools to assess and manage cases of DVA. It was not mandatory to use these tools. We did not set any targets and the offered tools could be used as the CMH professional deems necessary. Therefore, barriers and dilemmas regarding feasibility on the detection of DVA are comparable to those in the control group.

#### Sustainability of the BRAVE Intervention

According to the participants, a one-off training was not enough to ensure a full and effective implementation of the gained skills and knowledge about DVA in their daily routine. Almost all of the participants reported that the focus on DVA in the team in which they worked had been high in the first 2 to 3 months after the start of the BRAVE intervention. During the year of follow-up, however, their focus and knowledge steadily decreased. Some participants had implemented a routine question on DVA in their team meetings, which allowed them to remain focused on DVA in their patients. But most had not introduced such a structural inquiry, and their focus on DVA had dissipated. A comparison was made with their license to administer intramuscular injections: to prove their competence and have their license renewed, participants had to repeat the course in question. To maintain the effectiveness of a particular training, it should thus be repeated at least once a year and it should be obligatory. Participants also needed a recurrent stimulus to keep asking about DVA in their patients. While time-consuming surveys or forms were not desirable, short screening questions asked during intake, or a pop-up in the electronic patient file were found to be suitable ways of ensuring long-term sustainability. One participant preferred to make one person per team responsible for screening DVA in all patients. While most respondents agreed, some respondents also mentioned that screening for DVA is also a shared responsibility and that a whole team should be able to do so. Adding one person who is responsible to remind their peers to screen was a better option in their opinion. To maintain sustainability, it was also important for the team as a whole to have a positive attitude toward DVA screening, and for all members of the team to feel confident that their fellow team members did indeed screen for DVA within their caseloads. These elements were mentioned by participants who did not receive the BRAVE intervention as well.

### Mental Health Care Professionals Working in Intervention Teams or Control Teams

#### Acceptability and Feasibility of the Management and Referral of DVA

All participants saw DVA as an important and relevant topic. All participants also agreed that asking about DVA should be part of routine care and it is part of being a mental health professional to ask about it. However, many of them thought it was difficult to screen their patients for DVA and to manage the cases they detected. This mainly reflected a combination of practical and personal emotional barriers, dilemmas in the detection and referral of victims of DVA, and barriers in communication between DVA services and CMH services.

#### Barriers in the Detection of DVA

On a practical level, there appeared to be procedural obstacles. For instance, the questions asked to patients during the extensive intake for admission to a CMH team, are strictly protocolled. However, as this intake protocol does not cover DVA, professionals often forget to enquire about it. Participants suggested that this barrier could be resolved simply by adding DVA to the mandatory questions. Some participants said that they did not consider DVA a priority and that there mostly are more pressing issues demanding their attention. Adding a question about DVA to the protocolled questions would normalize talking about DVA—which could be helpful in further contact with the patient—and it could help and prioritize asking about DVA. If patients knew that these questions were part of standard procedures, they would not be considered intrusive or offensive.

##### Emotional barriers

On an emotional level, other participants reported that they could not find an opportune moment to discuss DVA. Doing so during the intake was considered premature and intrusive; building a therapeutic relationship was seen as a prerequisite to discuss DVA. However, not all participants agreed with this. Asking questions about sensitive topics is also part of being a professional in mental healthcare. Some participants argued, that during intakes, professionals asked many questions that are both sensitive and pragmatic. However, during routine care there is no standard format of questions. Uncertainty and not knowing what to do if a patient discloses being a victim of DVA play a major role in being apprehensive to ask about DVA.

“*But our routines don't have a fixed format. The question is also what our role is in all of this, because if you ask about DVA and find out that it's taking place, you also want to be able to do something about it. You want to have a response to it. So, I take this into consideration as well.”* [Control team, male, nurse]

Additionally, five participants from the control teams and one participant from the intervention teams who did not detect any signs of DVA during conversations with their patient, concluded that it was unnecessary to ask about it. Despite the emphasis given in the BRAVE intervention on active enquiry irrespective of physical signs of DVA, the same explanation was also provided by one participant who had taken the training. It was also the case that professionals usually opted to not discuss DVA, if the alleged perpetrator of violence was present when DVA was discussed with the patient, as this is deemed not to be safe either for the patient or the mental health professional.

##### Safety

Safety was also a possible issue when discussing DVA with patients, during home visits. All participants worked in CMH teams and made home visits, usually in pairs. To ensure their safety, they did not ask about DVA whenever they were not accompanied by a colleague. Potentially, the barriers described above could lead to delays and to not discussing DVA at all.

Participants were also hesitant to talk about DVA because they considered it taboo, much like talking about sexuality. With regard to being able to ask about a sensitive subject such as DVA, almost all participants said that mutual trust was necessary. In CMH care, mutual trust is not a given (22). It is often difficult for CMH professionals to engage patients who have little or no insight into their illness and their need for mental healthcare (23). As a result, participants reported their worries that discussing DVA might cause the patient to disengage and avoid future care.

“*You use the strength of the therapeutic alliance to assess whether or not you can discuss DVA. Sometimes this assessment tells you that if you discuss DVA now, you'll lose contact, and thus your grip on the whole situation.”* [Control team, male, nurse]
“*If they [patient] close the door on you, it's game over. There's nothing you can do anymore.”* [Control team, male, nurse]

However, when questioned about the actual consequences of discussing DVA with their patient, most participants reported that most of their patients had responded in a neutral manner, and contact remained unaffected. But some patients took offense, refused further healthcare, or asked to be transferred to a different CMH professional. This was especially prevalent in cases that also involved children.

#### Dilemmas in the Detection and Management of DVA

Finding a balance between these responsibilities often caused the participants moral and ethical dilemmas. For example, one participant referred to a female patient who prostituted herself in the sheltered housing where she lived. She was often raped by housemates and her customers, which negatively affected her mental and physical health. However, she refused to report these crimes to the police. On the one hand the participants wanted to protect their patient and keep her out of harm's way, that is, by reporting the sexual assault to the police without the patient's consent. On the other hand, they also wanted to respect her autonomy. The balance between protecting a patient and respecting their autonomy can be difficult. To be better able to handle the conflicting responsibilities and cope with the emotional burden, CMH professionals expressed the need for peer-to-peer consultation or a DVA consultant, which is not standard practice in mental healthcare institutions.

“*You just want to say that it affected you emotionally. But I know that if I said that in my team meeting, they'd just continue as if they hadn't heard. Nobody would ask how I felt.”* [Control team, female, social worker]

##### Patients refusing offered DVA services

Sometimes it so happened that participants detected DVA but the patient wanted no help for it. If the violence is not directly life-threatening—and all those involved were adults—victims of DVA cannot be mandated to accept help.

“*This happened in a case of mine—a patient who was being stalked and abused by her ex-partner in front of her –adult- children. Ideally, I'd have got them help right away, but the children didn't want help, the patient didn't want help… Which meant there was nothing I could do.”* [Control team, female, psychologist]

There is one exception to this rule, however. The presence of under aged children in a family gives the CMH professional a mandate to intervene to protect them from imminent harm, for example by requiring the DVA victims to accept or adhere to treatment, or otherwise to surrender their legal right to care for their children, or otherwise to surrender their legal right to care for their children.

“*Down the years Veilig Thuis wasn't involved, and nobody did anything about the children. But now they* **are** *involved, the penny dropped: my patient finally realized that she was about to lose her children and everything else.”* [Control team, female, social worker]

##### The patient as a perpetrator of DVA

Other dilemmas arose when the patient was the perpetrator and his or her partner was the victim. The training emphasized that discussing DVA with a potential perpetrator in the presence of the victim could lead to more violence or could prevent a victim to disclose DVA (10, 13, 21, 24). This means that where the patient is a perpetrator, the partner should be seen separately. This is not always possible due to healthcare professional-patient confidentiality; consent from the patient is required before any healthcare worker may discuss DVA separately with a partner. This consent is only needed if there is no risk of serious harm to the partner and/or if there are no underage children involved. A participant working in a control team dealt with this dilemma and tried to discuss DVA with the patient and partner simultaneously. However, research shows that discussing DVA with both a potential victim and perpetrator can be dangerous and therefore should be avoided at all times (21, 24).

“*I talked about DVA when [the perpetrator's/patient's] girlfriend was present. I asked if something had happened lately. She said no. But it's hard not to notice that he [the perpetrator/patient] is still in the room. He's a big guy. I think it's useful to discuss DVA, but under such circumstances, I'm not sure you'll get a truthful answer.”* [Control team Male nurse]

Usually, at the start of interviews with family, CMH professionals report that everything family says will be documented. However, partners sometimes told the CMH professional -before they could mention this disclaimer- that they were the victim of DVA and the patient the perpetrator, but urged the professional not to talk to the patient about it. This led to a dilemma, as all information regarding the patient has to be documented. In addition, all patients have the right to read all the information in their files. If the CMH professional would like to respond to the information provided and refer their patient -and perpetrator- for help, they would have to explain why they are referring. If the patient then asks who provided them with this information, the CMH professional has to share this with the patient. This was mentioned by the participants as dilemma, since this can be potentially dangerous for the partner.

“…*Or the partner is the victim and calls to say that DVA has taken place, but you mustn't talk to him about it. We do discuss this in the team. However, if you're the person treating the alleged perpetrator and the partner tells you to stay silent, you're stuck.”* [Control team, male, nurse]

Participants therefore felt that their abilities to provide the care they would want to provide were limited by their mandate and the rules on healthcare professional-patient confidentiality. As a result, CMH professionals could not always prevent the negative consequences of DVA.

#### Barriers to Managing and Referring Victims of DVA

In the focus in communication between CMH services and Veilig Thuis. As mentioned above, CMH professionals have to groups it became clear that participants experienced difficulties adhere to a professional code of conduct, part of which involves healthcare professional -patient confidentiality. By referring a patient against his or her will, they will breach that code and this requires a sufficiently good reason. It is often difficult to know when and how it is permissible to breach this confidentiality code. Participants expressed the need for an expert who can help with such legal questions. Although Veilig Thuis often requests medical information about a patient, healthcare professional -patient confidentiality means that it cannot always be given freely. Five participants remarked that those at Veilig Thuis were not always conversant with the rules and regulations governing the exchange of medical information. This perpetuated the communication difficulties and a discrepancy in expectations between Veilig Thuis and CMH professionals.

##### Barriers in Communication Between Mental Health Services and DVA Services

Even though Veilig Thuis assigned a case manager after a case of DVA was reported, participants criticized the manner in which this was interpreted and how they managed and provided care for their patients.

*“: My experience is that if Veilig Thuis gets involved, I end up on the sidelines—that they tend to take over my patient.”* [Control team, female, social worker]

As well as indicating the need for someone to coordinate all care and communications between all parties the involved, participants also said that if they reported a victim of DVA to Veilig Thuis, they were often not informed about the other parties and therapies to which the victim was then referred.

This could mean either that therapies were started that could negatively influence the patient's mental health, or that the parties involved all assume that another party is focusing on DVA.

“*Yes—whoever's responsible or in charge, there are so many players with so many different specialisms. Yes, we all want the same, but I regularly still see it going wrong. And that's sad.”* [Intervention team, female, nurse]

##### Dilemmas in diffusion of responsibility

Sometimes the outcome of these assumptions is that nobody does anything. Participants said they had to cross quite a high threshold before requesting help for a victim of DVA. Once they had reached that threshold, they expected the DVA services to view their case as urgently as they viewed it themselves.

“*I also expect DVA professionals to impose more. Once we had a woman in care with a husband and a baby who said a few times that she was being abused. We saw the signs, but each time it was just too little to prove the abuse. While there was also a baby … But, no, she [patient] didn't want us to treat her.”* [Control team, female, nurse]

As this was often not the case, however, frustration could follow, although some participants added that they knew their expectations were not always in line with reality.

“*If I speak for myself with regard to Veilig Thuis, whenever I report a case, basically I hope that the problem will now be resolved. But you know that it's not realistic.”* [Control team, female, psychiatrist]

##### Practical dilemmas

However, if a patient does accept help, and the situation requires the patient to move to a safe house, it is often difficult to find a place where the patient can go to. The places in shelters and safe houses are scarce and fully occupied most of the time.

When a CMH professional detects DVA, most want to intervene and stop the violence immediately. However, any combination of a patient's unwillingness to accept help, the CMH professional's and DVA services' limited mandate, and a shortage of places in safe houses leaves a CMH professional with few options to intervene.

## Discussion

### Main Findings

In this qualitative study, we explored whether it was both acceptable and feasible to provide an intervention aimed at increasing the detection of Domestic Violence and Abuse (DVA), whether the effect of such an intervention could be sustained, and how mental healthcare professionals managed with DVA in daily practice. This study was conducted in the context of a cluster randomized controlled trial. The trial did not demonstrate an effect of the training on DVA detection rate (*Ruijne et al., under review in journal of Interpersonal Violence*).

Despite the fact that all participants who participated in the focus groups found the intervention highly acceptable, topic relevant and important, participants had trouble maintaining the knowledge and skills they had acquired during the DVA training. This indicates that the effects of the intervention were not sustainable. The main reason for this was that their focus on DVA declined in the first few months after the training. This is consistent with previous literature. Allen et al. (25) and Trevillion et al. (3), found that to get professionals in healthcare to talk about DVA, it was important to design and implement standardized routine inquiries. Participants agreed with these findings, and said that asking about DVA should be part of their own standard, routine enquiry. They also stressed that a training such as BRAVE should be repeated once a year.

The intervention required CMH professionals to ask their patients about DVA on a regular basis. This proved to not be feasible for participants. Both participants from the intervention teams as well as participants from the control teams provided possible explanations for this. The first possible explanation participants provided for not asking about DVA was a fear of disrupting the therapeutic alliance and a fear of symptoms increasing in patients by asking about DVA, especially if there are underage children involved. Participants found it difficult to know when and where to ask, regardless of receiving the intervention or not. The participants struggled with their need to protect victims from continued abuse and violence, which might involve breach of healthcare professional -patient confidentiality in the case of adult victims vs. patient autonomy. Parallels could be drawn with asking about trauma in Dutch SMI patients. For a long time Dutch professionals did not ask about trauma because this was taken to mean that the patient will show more symptoms, which in turn could lead to more medication or even hospitalization (26). However, the opposite view is now accepted. Patients who are asked about and treated for trauma show a significant decrease in symptoms compared to patients who have not been treated (27, 28). In the article by Trevillion et al. (29) professionals also expressed their fear of offending a patient when they asked about DVA. In the same article, however, service users said they wanted professionals to talk about DVA, as they believed it would encourage disclosure. Similar results were found by Feder et al. (30). They found that it was important to service users that professionals not only adopted a supportive and non-judgmental attitude toward DVA, but also that they provided ongoing support. It is important for professionals not to hold back on enquiry until they feel the time is right. Withholding could play upon a victim's anxieties about the stigma and shame of the abuse, while routine enquiry could instead help relieve them. Additionally, asking about DVA would also show that the professional understands how common DVA is and can help if a victim chooses to disclose, which is in accordance to the NICE (National Institute for Health and Care Excellence) guidelines (31). Since managing DVA in patients can also be emotionally challenging for CMH professionals, mental health institutions should provide peer-to-peer support for CMH professionals.

A second explanation could be the safety of DVA victim and CMH professionals. If the patient is a perpetrator, this adds complexity to DVA management. Less research has been done about patients being perpetrators and the best course of action in standard mental healthcare. However, this theme consistently emerged during the focus group discussions and caused many dilemmas in daily practice (32). While talking about DVA in the presence of a possible perpetrator can sometimes lead to dangerous situations for the victim (13, 24, 33), participants indicated that it was not always possible to talk to a possible victim of DVA in private, especially when the patient was a perpetrator and their partner a victim. Because DVA was a taboo topic for some participants, they did not feel comfortable discussing it with patients if they were alone in the patient's home. This feeling stemmed mainly from previous experiences of aggression, from not feeling skilled enough to discuss it, or from lacking the ability to discuss it.

A third explanation could be the participants' lack of mandate to intervene, accompanied by the frustration they often felt when they had to manage DVA in their patients. If adult DVA victims want to receive help, they have to be willing to accept it. Unfortunately, this doesn't take into account the nature of coercive controlling DVA and the fear many patients have that violence will escalate if they leave—as indeed it can, evidenced by the fact that DVA homicide is more likely at the point of or after leaving a DVA perpetrator. Participants mentioned that they can press charges for a patient, or for the partner of that patient, only if they feel that his or her life is in danger and if they feel able to prove the victimization. As professionals cannot force a patient to press charges or to accept help for DVA, moral dilemmas, frustration and a feeling of defeat can follow—and this may, unconsciously or otherwise, lead some professionals not to ask about DVA at all.

### Limitations

Due to the reorganization and introduction of Veilig Thuis at the start of the BRAVE study, Veilig Thuis was unable to work closely with CMH professionals. As a consequence, we found that the cooperation between DVA services and mental health services needs to be improved. Participants feared that the lack of communication and coordination in the management of DVA cases would ultimately lead to substandard care, which might lead in turn to more severe physical and mental consequences, and/or aggravation of the violence to which a patient was exposed. It was also clear that it was important to provide access to a DVA expert for questions and help in management of DVA, and more attention should be paid to the emotional well-being of the mental health professionals who dealt with cases with DVA.

This study was conducted during a nationwide reorganization of DVA services. Institutional reorganizations are very common in the healthcare and social sector, making it difficult to provide a consistent approach in providing care, and impacted on our intervention not being long-lasting. Although our sampling continued until the data was saturated, it should be noted that a majority of our sample consisted of nurses. While, in theory, psychiatrists or psychologists may have given different views on the problem of DVA in psychiatric patients, this was not the case in this sample, which was representative of the general composition of an average CMH team (34), suggesting a generalizability of the results.

## Conclusion and Clinical Implications

In recent years, DVA has gained more attention in the domain of clinical work. Its detection nonetheless remains low, and few interventions have attempted to increase it. Our findings highlight the importance of a multidimensional intervention that focuses not only on CMH professionals but also on institutional inputs such as setting up peer-to-peer support for CMH professionals, and using prompts in patient files to remind professionals to ask about DVA. When training is part of an intervention, it should be inspirational and informative. Importantly, it should also be recurrent. It is equally important to focus on how to ensure successful implementation, a matter that requires a good understanding of the local contexts in which the services operate, ensuring the proper functioning of DVA referral sites, and ensuring extensive communication and collaboration between CMH institutions and DVA services. An important starting point could be to actively facilitate enquiries about DVA and making them part of the routine clinical enquiry. Finally, as it is also important not to underestimate the possible impact on the CMH professional of working with victims of DVA, these professionals should be given space to express their concerns and discuss cases within the organizations where they work.

## Data Availability Statement

The data that support the findings of this study are available on request from the corresponding author, RR. The data are not publicly available due to their containing information that could compromise the privacy of the research participants.

## Ethics Statement

The studies involving human participants were reviewed and approved by Medical Ethical Committee at the Erasmus University Medical Center, MEC-2015-409. The participants provided their written informed consent to participate in this study.

## Author Contributions

RR prepared the topic list, included the participants, collected the data, and analyzed the data and prepared the manuscript. AK prepared the topic list, and analyzed the data and prepared the manuscript. KT provided feedback on the topic list and on the manuscript. CG, MG, MZ, SB, LH, and CM all provided feedback on the manuscript throughout the whole process of preparing the manuscript. All authors contributed to the article and approved the submitted version.

## Conflict of Interest

The authors declare that the research was conducted in the absence of any commercial or financial relationships that could be construed as a potential conflict of interest.
