# Supplementary material for: Assessing the Acceptability, Feasibility and Sustainability of an Intervention to Increase Detection of Domestic Violence and Abuse in Patients Suffering From Severe Mental Illness: A Qualitative Study
Source: Front Psychiatry. 2020 Oct 28;11:581031. doi: 10.3389/fpsyt.2020.581031 (PMC7655979; doi:10.3389/fpsyt.2020.581031)
Supplement: Supplementary file 1 [file Data_Sheet_1.docx]

Supplementary Material for: Assessing the acceptability, feasibility and sustainability of an intervention to increase detection of domestic violence and abuse in patients suffering from severe mental illness: a qualitative study

# Brave study topic guide

We created a study topic guide to help to focus on specific themes during the focus groups. We added underscores for themes/subjects that were added during the study period or after a focus group session.

**BRAVE study topic guide:**

**Focus group interviews with community mental health professionals enrolled in the BRAVE study**

**Introduction**

In 2015 we started a research project to raise awareness and increase detection of domestic violence and abuse in psychiatric patients. As part of this research project we gathered you here to ask you questions on domestic violence, your experiences with domestic violence in your patient population, and what your role is in the care for domestic violence and abuse. If you received the BRAVE intervention we would like to know your opinion on this intervention and if this changed the way you provide care for your patients. We also want to know what you think about the current protocol for domestic violence and abuse in psychiatric patients and what your improvements for this protocol are and how you would make sure these improvements are followed. There is no right or wrong answer.

**Role of participants:**

1. Could you start by telling me a little about yourself and your experience with domestic violence?

**General question about domestic violence:**

1. **What do you think of when you think of domestic violence and abuse?**

Probe for:

- Their own definition of domestic violence
- Opinions about victims/perpetrators of domestic violence
- Stereotypes/assumptions

1. **You received a training on domestic violence and abuse what can you recall from that training?**

- Probe for:
- Good points on the training
- Improvement on the training
- Expectations on the training, did they come true yes or no?
- Was it useful? Yes/no and why?
- Practical issues?
- Opinion about the trainer and what makes an ideal trainer?
- What can you recall from the training

1. **A. How do you manage DVA in your patients?**

- Probe for:
- Do you ask about domestic violence? Yes or No and why?
- How does a conversation about domestic violence look like?
- What makes you ask about domestic violence?
- When will you not talk about domestic violence?
- *For participants who received the training:*
- - Do you do anything differently compared to before receiving the intervention?
- - Did the training help you in the daily routine of caring for a patient? In what way? If not, what would have helped?

**4.B. How do patients react?**

Probe for:

- What is your experience with asking about domestic violence. Do these differ between genders?
- Are there patient related factors will make you not ask about domestic violence?
- Did asking about domestic violence ever have consequences, negative or positive?
- *For participants who received the training:*
- - How do patients react when you talk about domestic violence?
- - How do patients react when you use the Meldcode Protocol?
- - Did patients get back to you on the subject of domestic violence and abuse?
- - Did patients disclose domestic violence spontaneously?
- - Does it influence your professional-patient relationship, both bad and good experiences?
- - Did the training help in getting the patient the proper care more quickly?

**4.C. Does your team discuss DVA?**

Probe for:

- How often?
- When do they discuss domestic violence, when do they not?
- What do you consider a good frequency?
- *Participants who received the training*
- Did your team do things differently regarding DVA? In what way?
- Is there still a difference noticeable?
- What do you think will happen with the topic of DVA now this research project is finished?

1. **What is your experience with the available services for domestic violence and abuse?**

- Probe for:
- Experiences with DVA services.
  - Explicitly ask about positive experiences as well
- How is your experience with communication with DVA services?
- How would you want to take care of your patient in an ideal world?
- What would you like to see differently?
- *Participants who received the training*
- Do you approach DVA services differently?
- Does the training make you better equipped to manage DVA, if yes in what way? If no, why not?

**End of interview**

1. **Is there anything you would like to add?**

# Analysis process

Due to ethical restrictions we are unable to show our entire code book. However, we have opted to show an example of our coding system using the data we used in our manuscript. The coding of qualitative data is a circular process. To give more insight in how we exactly coded all our data we will describe a linear process. However, this process describes in broad, general steps how the data was coded. Before we started coding all the transcripts, we established our main frameworks and broader concepts: the acceptability, feasibility and sustainability of the BRAVE intervention and the acceptability and feasibility of DVA management and referral in general. Supplementary figures one, two and three show an example of our coding system. First, we started with reading all the transcripts and coding all text relevant to our main framework. These predetermined frameworks or overall broad themes were later used as frames to answer our research question. In this process we selected and coded them accordingly. Simultaneously, we used first-order coding for interesting topics which were not directly related to our predetermined framework and made notes on these topics . Second, after initial coding we read the coded texts again, dividing these larger portions of text into units of 5-line blocks of text, which is (approximately) half a paragraph , each with a less broad theme such as on the acceptability of the intervention all text about the trainers, the content or facilities. In this step we subthemes emerged and were, if possible, placed in an pre-existing framework. All specific open codes were placed under a more broad theme. In this step we also assessed the inter-rater reliability. To calculate the inter-rater reliability we used units of 5-line blocks of text and assessed whether the text coded for a theme and which theme (acceptability, feasibility or sustainability). Coding for subthemes, emerging themes, valence etcetera was subject for consensus discussion. Third, we further specified the themes, looking for common subthemes to group together such as advice on improvement of the BRAVE intervention. We then used inductive analysis, and frame analysis to identify sub themes, link themes, draw conclusions or identify explanations for our initial research questions. This process of inductive analysis and frame analysis continued until we were satisfied with the specificity of the data and until we found our initial research question to be answered and the emerging themes to be sufficiently covered. The fourth step was to report our results.


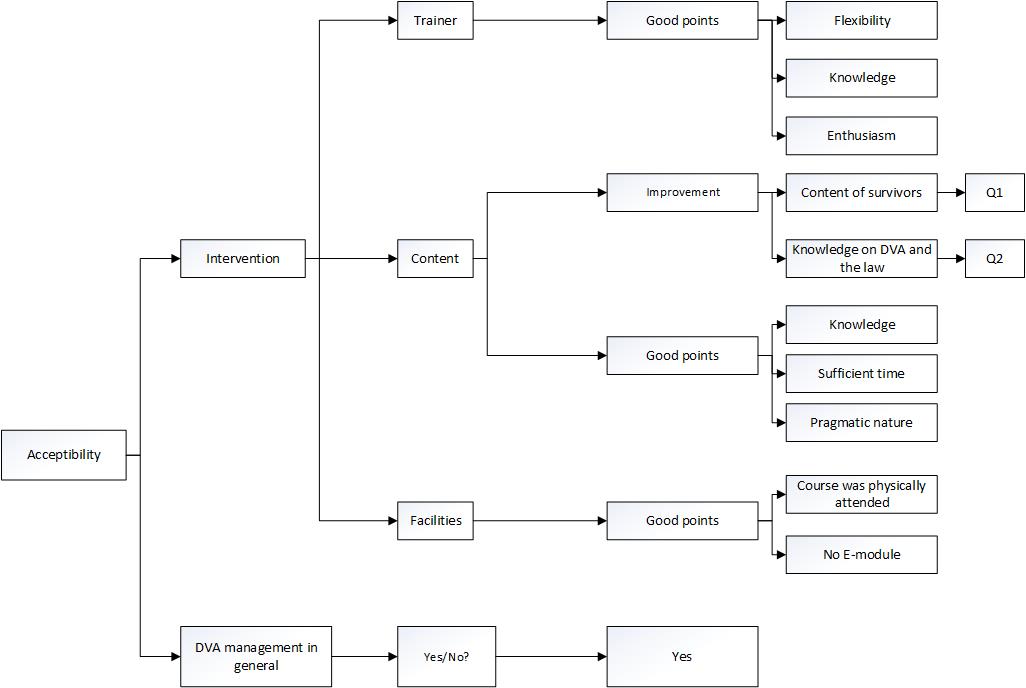


**Supplementary figure 1. Code book on the theme acceptability. Q1, Q2= quotes are described below**

**
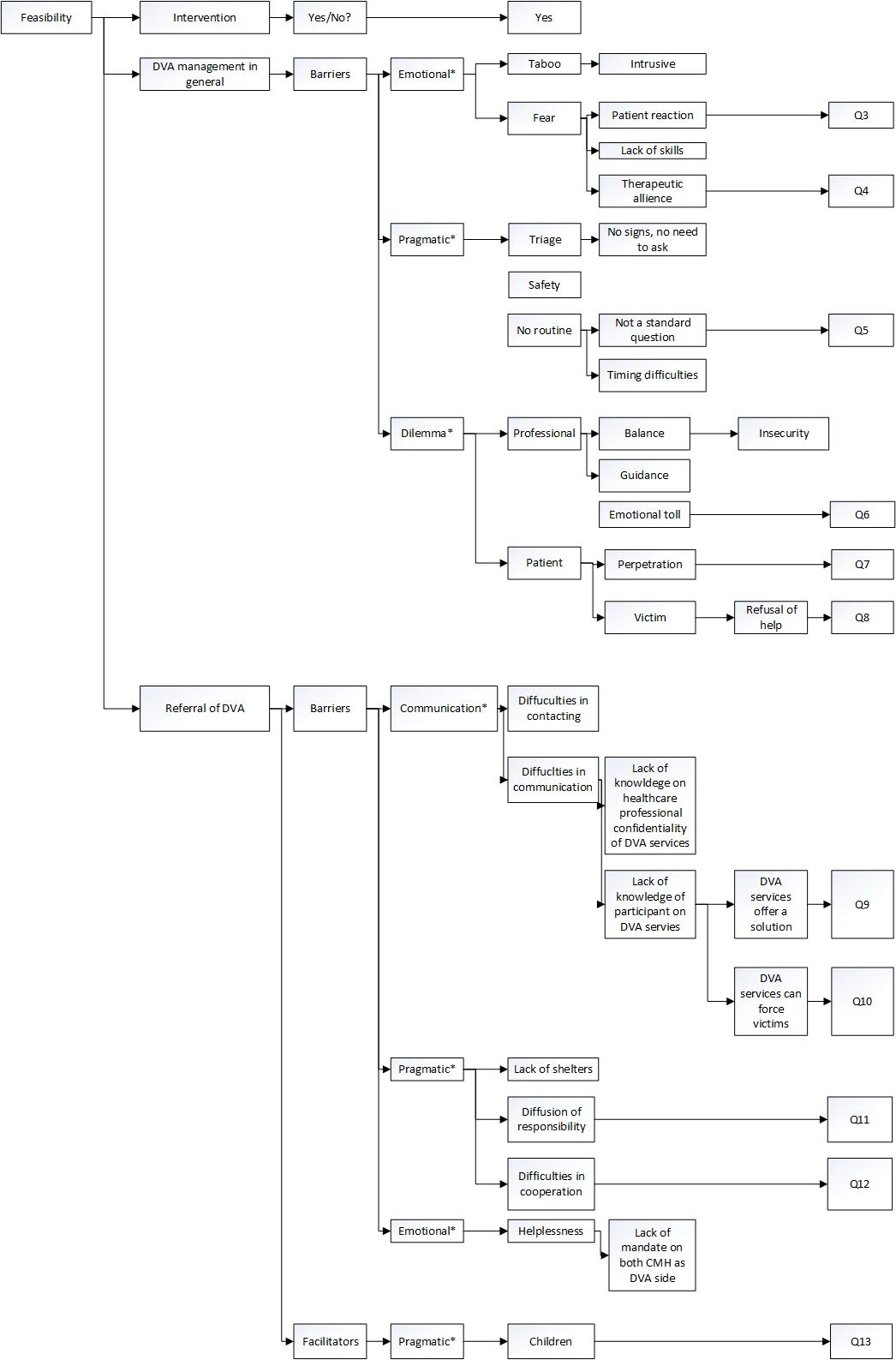
**

**Supplementary figure 2. The code book of the feasibility of the BRAVE intervention and DVA management in general. *= themes derived from data. Q=quotes and are described below**

**
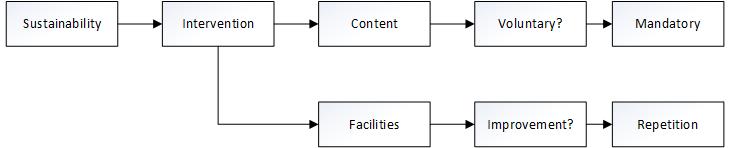
**

**Supplementary figure 3. The code book of the feasibility of the BRAVE intervention**

**Q1:** **Intervention team, male, nurse (on the training:** *“Hearing about the victims’ experiences helped me a lot. During the training on DVA, one of the victims told the story of the abuse she’d endured from her mother. That had a real impact on me—it made me more alert, and it made me think.”*

**Q2:** **Control team, female, social worker:** *“I now think I’d like to have heard more about healthcare professional -patient confidentiality and your duty to provide proper care. The training covered it a bit, but there should have been more about it.”*

**Q3: Control team, male, nurse: *“****If they [patient] close the door on you, it’s game over. There’s nothing you can do anymore.”*

**Q4: Control team, male, nurse:** “*You use the strength of the therapeutic alliance to assess whether or not you can discuss DVA. Sometimes this assessment tells you that if you discuss DVA now, you’ll lose contact, and thus your grip on the whole situation.”*

**Q5: Control team, male, nurse: *“****But our routines don’t have a fixed format. The question is also what our role is in all of this, because if you ask about DVA and find out that it’s taking place, you also want to be able to do something about it. You want to have a response to it. So, I take this into consideration as well.”*

**Q6: Control team, female, social worker*:*** *“You just want to say that it affected you emotionally. But I know that if I said that in my team meeting, they’d just continue as if they hadn’t heard. Nobody would ask how I felt.”*

**Q7: Control team Male nurse***: I talked about DVA when [the perpetrator’s/patient’s] girlfriend was present. I asked if something had happened lately. She said no. But it’s hard not to notice that he [the perpetrator/patient] is still in the room. He’s a big guy. I think it’s useful to discuss DVA, but under such circumstances, I’m not sure you’ll get a truthful answer.”*

**Q8: Control team, female, psychologist***:” This happened in a case of mine – a patient who was being stalked and abused by her ex-partner in front of her –adult- children. Ideally, I’d have got them help right away, but the children didn’t want help, the patient didn’t want help… Which meant there was nothing I could do.”*

**Q9: Control team, female, psychiatrist:** *“If I speak for myself with regard to Veilig Thuis, whenever I report a case, basically I hope that the problem will now be resolved. But you know that it’s not realistic.”*

**Q10: Control team, female, nurse**: *“* *I also expect DVA professionals to impose more. Once we had a woman in care with a husband and a baby who said a few times that she was being abused. We saw the signs, but each time it was just too little to prove the abuse. While there was also a baby … But, no, she [patient] didn’t want us to treat her.”*

**Q11: Intervention team, female, nurse: *“*** *Yes—whoever’s responsible or in charge, there are so many players with so many different specialisms. Yes, we all want the same, but I regularly still see it going wrong. And that’s sad.”*

**Q12: Control team, female, social worker:** “***:*** *My experience is that if Veilig Thuis gets involved, I end up on the sidelines – that they tend to take over my patient.”*

**Q13: Control team, female, social worker***: “Down the years Veilig Thuis wasn’t involved, and nobody did anything about the children. But now they* **are** *involved, the penny dropped: my patient finally realized that she was about to lose her children and everything else.”*

# Supplementary material table 1.

| Table 1. Key themes and sub-themes discussed by focus group participants | | |
| --- | --- | --- |
| **Intervention teams trained in managing DVA** | | |
| **Key themes** | **Sub-themes** |  |
| Acceptability of the BRAVE training | Content of the course | - There was time available to discuss dilemmas and personal experiences during the course and practice situations - The training course was sufficient in providing knowledge on DVA - The course was pragmatic in nature - Knowledge and practical information on healthcare professional -patient confidentiality and confidentiality should be incorporated in the training course - Real life experiences of DVA survivors are valuable and inspire change in behavior |
|  | Training facilities | - A course on DVA should be physically attended - E-modules are not seen as beneficial |
|  | Trainer | - The trainer had sufficient knowledge on both community mental health teams and DVA services - A trainer should be able to enthuse and motivate |
| Feasibility of the BRAVE intervention | Training course | - Attending the training course was feasible |
| Sustainability of the BRAVE intervention | Training course | - The training course was only sustainable if it would be a recurrent course - The training course should be obligatory |
| **Intervention and control teams** | | |
| **Key themes** | **Sub-themes** |  |
| Feasibility of the management and referral of DVA | Barriers in the detection of DVA* | - Routine enquiry about DVA is not part of the intake protocol - DVA could not be seen as the most pressing problem and is therefore not always a priority. - Lack of a therapeutic alliance makes it difficult for CMH professionals to ask about DVA - CMH professionals find it difficult to find an appropriate time to talk about DVA with their patients - Asking about DVA is seen as intrusive - If no DVA signals are seen, CMH professionals do not feel the need to ask - Fear and feeling a lack of abilities and skills to manage DVA if it would be detected - Asking about DVA could lead to unsafe situations for the CMH professional and the victim - -DVA is considered a taboo subject - A patient will refuse mental healthcare if you ask about DVA |
|  | Dilemmas in the detection and management of DVA* | - It is difficult to find a balance between patient autonomy and the health care professionals’ responsibilities for the patient’s welfare. - Lack of a familiar DVA consultant to ask questions regarding DVA - There is a lack of focus on the emotional wellbeing of health care workers - The patient as a perpetrator of DVA leads to dilemmas in confidentiality between patient and healthcare professional - CMH professionals find themselves lacking mandate when a patient refuses to accept help for DVA |
|  | Barriers to managing and referring victims of DVA * | - DVA services are unfamiliar with the CMH services’ rules and regulations , such as healthcare professional -patient confidentiality - DVA services at times do not seem to communicate with the CMH professional and take over the care for the patient - Lack of a central person responsible for all parties involved in the management of DVA - DVA services are sometimes difficult to contact - Participants’ unfamiliarity with the rules and regulations of the DVA services |
|  | Expectations of DVA services* | - DVA services can offer direct a solution to the problem of DVA - DVA services can force victims to accept help - Lack of shelters - Lack of mandate for both DVA services as CMH professionals |
| * themes derived from the focus group discussions, DVA= domestic violence and abuse, CMH = community mental health teams | | |
